# Simulation Research on Continuous Concrete Beams Reinforced with External Prestressed CFRP Tendons

**DOI:** 10.3390/ma15165697

**Published:** 2022-08-18

**Authors:** Ning Duan, Ji-Wen Zhang, Jun Cheng

**Affiliations:** Department of Civil Engineering, Southeast University, Nanjing 210096, China

**Keywords:** carbon fiber composite plastic (CFRP), finite element analysis, prestress, bending moment

## Abstract

This paper examines the effects of different loading patterns on the static characteristics of continuous concrete beams reinforced with external prestressed carbon fiber-reinforced polymer tendons (EPCFRPT) and qualitatively analyzes the results for two continuous concrete beams: SB-1 under symmetrical loading, and SB-2 under asymmetrical loading. Then, a finite element analysis model is introduced and calibrated by tests conducted at Southeast University and data collected from a literature review. Based on the FEA model, the initial prestress, cross-section area, and eccentricity of CFRP tendons as well as the steel reinforcement configuration were selected by a parametric study. The results indicated that the initial prestress and tendon cross-section area had the most influence on the tendon stress increment and the secondary bending moment of the middle support, while the reinforcement distribution and eccentricity of the tendons had little effect. The secondary bending moment had a linear positive correlation with the stress increment of tendons. These results allow a simplified equation for calculating the external load bending moment amplitude coefficient at ultimate to be proposed.

## 1. Introduction

External prestress technology has been widely used in concrete structures, and is the most effective method for reinforcing existing structures [1,2]. Steel strands are widely used as external prestress tendons in civil engineering. However, the use of steel strands in structural strengthening engineering faces durability issues [3]. Fiber-reinforced polymer materials can be used to replace steel strands for structural strengthening, as they have the characteristics of light weight, high strength, fatigue resistance, corrosion resistance, and good chemical and non-magnetic properties [4,5].

The internal force redistribution of external prestressed concrete continuous beams is the result of external load bending moment redistribution in combination with secondary bending moment changes [6,7,8]. The CEB-FIP [9] recognizes that the effects of secondary bending moments should be considered in both the service phase and the ultimate load-carrying capacity state. Reasonable consideration of moment redistribution is of great significance in the analysis and design of continuous beams with high flexural strength. The initial secondary beading moment has an effect on the stiffness of the continuous beam section [10] due to the prestress tendons limiting plastic hinge rotating in the middle support, while the restraining action exists until failure [11]. According to Tiejiong Lou [12], the secondary moment is proportional to the tensile stress of the prestress tendons in the nonlinear phase. The secondary bending moment increases as the stress on the prestressed tendons increases. The research of Lu Zhitao [13] showed that the influence of the secondary bending moment must be considered in the load phase. In the ultimate limit state for a prestressed statically indeterminate structure with a high reinforcement ratio, the secondary bending moment must be considered.

The analysis of external non-bonded prestressed concrete continuous beams is much more complicated compared with external bonded prestressed concrete continuous beams [14]. Allouche [15,16] determined a simplified analytical method by assuming that the beam remains elastic before cracking and forms a plastic hinge during failure. Their research results laid a foundation for later studies by Harajli [17,18] on the influence of the external prestressed steel bar arrangement on concrete continuous beam performance, leading to the establishment of a model based on the incremental deformation method. Tiejiong Lou [19] performed a numerical simulation study on the bending behavior of concrete continuous beams with external prestressed steel bars. Abbas Tajaddinil [20,21] studied the bending moment redistribution performance of reinforced concrete continuous T-beams strengthened with FRP and proposed a method for calculating the bending moment redistribution.

The object of this article is to study the effect of external prestressed tendons on the static behavior of continuous beams, and especially on the redistribution of bending moments, which is one of the key problems when using external prestressed CFRP-reinforced concrete continuous beams. To this end, we highlight the deformation characteristics, ultimate load, and stress redistribution of continuous beams with external prestressed CFRP tendons under symmetrical and asymmetrical loading.

## 2. Testing Setup

### 2.1. T-Beam Design

**Figure 1:**
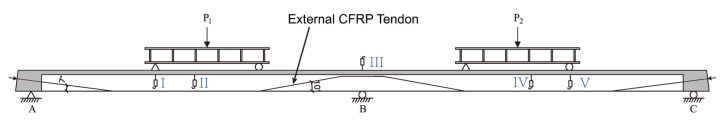
Two-span continuous concrete T-beams: I is the control section of AB, II is the mid-span section of AB, III is the mid-span section of the beam, IV is the mid-span section of BC, and V is the control section of BC span.

Two prototype two-span continuous concrete T-beams with cross-sections and tendon layouts are shown in Figure 1. In this paper, we examine the effects of load on the static performance of specimens such as this one.

**Figure 2:**
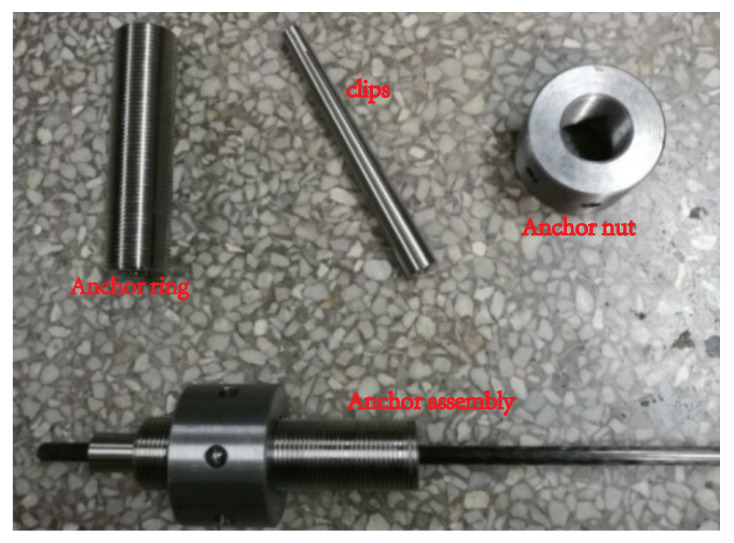
Integral clip-type anchor.

The total length of each beam was 6.7 m, of which each span was 3.2 m. Each beam was 200 mm high and the span-to-depth ration was 16. Two deformed steel beams (ϕ14) were placed in the tension zone and four steel bars (ϕ8) in the compression zone. Steel stirrups (ϕ8) were spaced at 80 mm along the length of the beam. Two domestic CFRP prestressed tendons (ϕ8) were arranged outside the continuous beam. In order to keep the line type of the prestressed CFRP tendons consistent with the shape of the bending moment diagram of the external load as well as to reduce the secondary effect caused by the eccentricity of the prestress tendon in the mid-span, six steering blocks were set on each side along the beam axis. The bending angles of the CFRP tendons were 7° at the four inner-span steering blocks and 10° at the two middle support steering blocks. Self-developed integral clip-type anchors, shown in Figure 2, were used to anchor the external CFRP bars.

The designed equivalent reinforcement ratio is

(1) $$r=\frac{A_{s}+A_{p}f_{fu}/f_{y}}{bh_{0}}=2.9\%$$

where $A_{s}$ and $A_{p}$ are the area of the longitudinal reinforcement and CFRP tendons, respectively, $f_{fu}$ is the ultimate tensile strength of the CFRP tendons, $f_{y}$ is the yield strength of the steel bars, *b* is the width of the specimens, and $h_{0}$ is the height of the effective compression zone. The prestress degree is

(2) $$PPR=\frac{A_{p}f_{pe}}{A_{p}f_{pe}+A_{s}f_{y}}=0.407$$

where $f_{pe}$ is the designed effective prestress and $f_{pe}=900$ MPa.

### 2.2. Materials

We used HRB400 grade deformed steel bars with an average yield strength of 426 MPa. The ultimate tensile strength and elastic modulus of the external CFRP tendons were 2020 MPa and 145 GPa, respectively. The properties of the CFRP tendons and deformed steel bars were tested according to Chinese codes GB/T 228.1-2010 (2010) [22] and GB/T 30022-2013 (2013) [23]. The test results involving these properties are listed in Table 1.

Commercial concrete was used in the tests. In accordance with reports from concrete suppliers, the concrete mix ratio was cement:medium sand:gravel:water at 1:1.07:2.18:0.43 by weight. River sand was used. The amounts of the different ingredients in the concrete are shown in Table 2. The cement was ordinary Portland cement 42.5. Its chemical composition included CaO, SiO_2_, Al_2_O_3_, and Fe_2_O_3_ with mass percentages of 62%, 21%, 5%, and 3%, respectively, tested following Chinese code GB175-2007 [24]. When pouring the continuous beam, three cube concrete blocks were reserved for measuring the 28-day cube compressive strength of concrete, $f_{cu}$, according to Chinese code GB/T 50081-2016 (2016) [25]. The concrete used in each specimen was as listed in Table 3.

### 2.3. Testing Procedure

Test beam SB-1 was in asymmetrical loading mode, with the two spans synchronously loaded to P1=40 kN. The AB span maintained a constant load of 40 kN and we continued to load the BC span continued until SB-1 sustained damage. By contrast, test beam SB-2 was in symmetrical loading mode; the two spans were synchronously loaded until SB-2 failed. Aside from the loading modes, the remaining loading criteria for SB-1 and SB-2 were the same. Load control was first applied to control the loading process before yielding, and the loading was increased in increments of 10 kN. When cracking was imminent, the increment in loading was properly reduced to identify the precise cracking load. After the test beam had yielded, displacement control was applied to control the loading process. The relevant data were obtained after three minutes, when each step of loading had been completed, in order to ensure accuracy. Displacements of the loading point, mid-span, and support were measured by a displacement meter (Figure 1).

## 3. Test Results and Discussion

### 3.1. Failure Type

**Figure 3:**
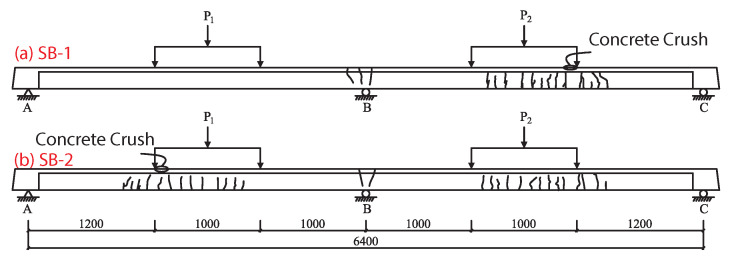
Distribution ofc oncrete cracks in continuous beam: (**a**) SB-1, (**b**) SB-2.

The final failure modes of continuous beams SB1 and SB2 are both flexural failure. The distribution of concrete cracks are shown in Figure 3.

SB-1 Failure: before the loading reaches 40 kN, the beam is in the elastic stage. When the load reaches 40 kN, the concrete at the lower edge of the BC span control section and the upper edge of the middle support section simultaneous cracked in tension, and the beam enters the nonlinear stage. Subsequently, the load on the AB span remains unchanged, the BC span continues to be loaded, the deflection growth rate of the BC span beam body is accelerated, and the growth rate of the external prestress CFRP reinforcement stress is accelerated. When the BC span is loaded to 98 kN, the BC span control section and the tensile reinforcement of the middle support section yield at the same time, then the BC span deflection and the external CFRP reinforcement stress enter a stage of rapid growth. When the load reaches 114 kN, the concrete in the upper edge compression zone on the inner side of the BC span control section is crushed, and the continuous beam losses its carrying capacity.

SB-2 Failure. Before the load reaches 45 kN, the beam is in the elastic stage. When the load reaches 45 kN, the lower edge of the AB and the BC span control section, as well as the upper edge of the middle support section, simultaneously crack due to tension stress. The beam then enters the nonlinear stage. As the two spans continue to be loaded synchronously, the growth rate of the deflection of the continuous beam and the stress of the external prestressed CFRP bars both accelerate. When the load reaches 80 kN, the tensile reinforcement of the middle support section yields, and there is a significant moment redistribution phenomenon. As loading continues to 105 kN, the tensile reinforcement of the AB span and BC span control section yields, then the mid-span deflection and external CFRP reinforcement stress enters a stage of rapid growth. When the load reaches 127 kN, the concrete in the compression zone on the inner side of the AB span control section is crushed, and the continuous beam loses its bearing capacity.

### 3.2. Load–Displacement Relationship

**Figure 4:**
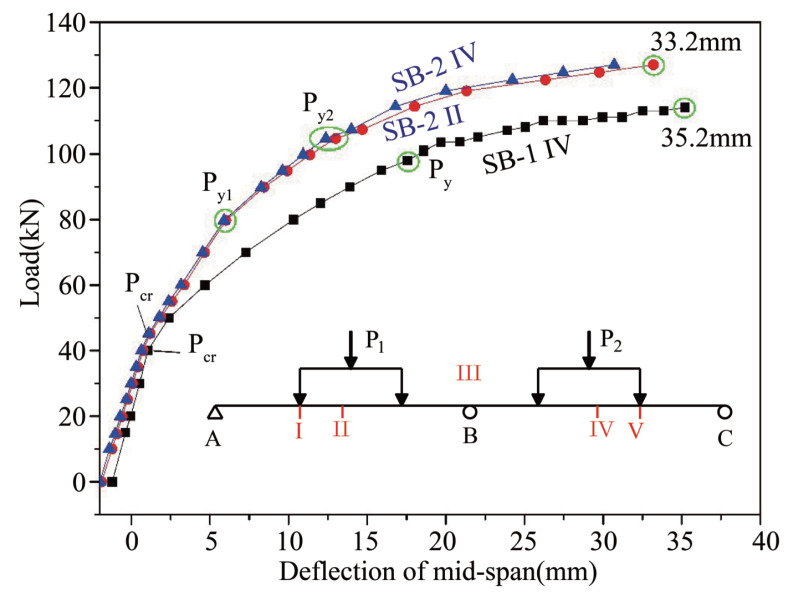
Load–deformation curves of SB-1 and SB-2 beams. P1 and P2 are applied loads, I, II, III, IV, and V are specific points along the beams where beam behavior under loading was analyzed, $P_{cr}$ is the cracking load, $P_{y}$ is the load at which the steel reinforcement yielded, and $P_{u}$ is the ultimate load at beam failure.

Figure 4 demonstrates the relationship between the load and the mid-span deflection of the test beams. The whole response process of the continuous beams under loading can be divided into three stages, irrespective of loading modes. These stages consist of the elastic stage before the test beam cracks (i.e., the critical load $P_{cr}$ is not reached) and two nonlinear stages in which the beam deflection increases rapidly due to reduced stiffness of the beams, which results from the concrete in the tensile zone cracking (i.e., $P_{cr}$ is reached) and the tensile steel yielding at the control section ($P_{y}$ is reached).

However, several differences are apparent between the different load–deformation curves. First, the tensile steel rods at the V and III sections of SB-1 (Figure 1) yielded simultaneously at a yield load of 98 kN. However, in SB-2 the tensile steel rods at section III first yielded at 80 kN, while the tensile steel rods at sections I and V yielded at 105 kN. This meand that two yield points, $P_{y1}$ and $P_{y2}$, are included on the load–deformation curve of SB-2. Second, the ultimate load of SB-1 was 114 kN and the ultimate deflection was 35.2 mm, which was approximately 1.1% of each span length. The ultimate load of SB-2 was 127 kN and the ultimate deflection was 33.2 mm, which was approximately 1% of each span length.

Thus, for a two-span continuous beam, the ultimate load under symmetrical loading (SB-2) was higher than that under asymmetrical loading (SB-1). During symmetrical loading of SB-2, the tensile steel rods at the mid-support section (section III) yielded before the tensile steel rods at the mid-section of each span (sections IV and V). Conversely, during asymmetrical loading of SB-1, the tensile steel rods at the mid-support section (section III) and the mid-section of the BC span (section V) yielded almost simultaneously.

The load–displacement curve of the external CFRP-reinforced concrete continuous beam in this paper is similar to the load–deflection curve of the external prestressed steel cable continuous beam obtained by Tan [26]. The direct cause is the cracking of the concrete and the yielding of the tensile steel bar.

### 3.3. Moment Redistribution

In addition to satisfying the conditions of static balance, statically indeterminate concrete structures need to satisfy the conditions of deformation coordination in order to obtain structural response under load. For statically indeterminate concrete structures, the relationship among the internal forces in different sections does not follow a linear elastic state owing to the inelastic properties of such structures after they crack. This phenomenon is called internal force redistribution, and is usually reflected in the moment redistribution. Many approaches have been proposed that consider internal force redistribution. One of these is the method of the coefficient of moment redistribution, which modulates the value of the moment calculated by the elastic theoretical method to ensure that it is close to the actual forces of the structure. In this paper, this method is applied to analyze the effects of different loading patterns on the internal force redistribution of SB-1 and SB-2. The moment redistribution coefficient, β, is usually calculated by the function [27]

(3) $$\beta =\frac{M_{e}-M_{t}}{M_{e}}\times 100\%$$

where $M_{e}$ is the theoretical value and $M_{t}$ is the experimental value of the moment.

**Figure 5:**
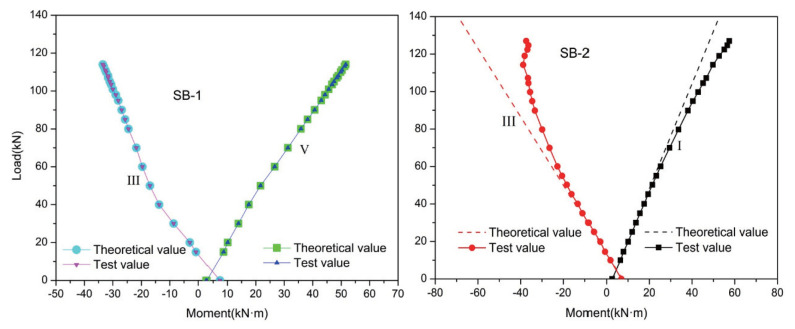
Load–moment relationship.

Figure 5 shows the moment redistribution at control section V of SB-1 and control section I of SB-2, as well as at support section III of both SB-1 and SB-2. It shows that the theoretical and experimental values of the moments at all control sections are non-zero when the external load is zero. This result is obtained because of the secondary bending moment that arises following tension in the external prestressed CFRP tendons. Before SB-1 and SB-2 cracked, the experimental value was almost coincident with the elastic theoretical value, which indicates that no moment redistribution had occurred. After SB-1 cracked, the theoretical value only slightly differed from the experimental value; $\beta_{III}$ and $\beta_{V}$ were only 3.7% and −1.0%, respectively. However, when SB-2 cracked, the test value deviated from the theoretical value, and the degree of deviation increased with the increase in load. By the time SB-2 had lost its bearing capacity, $\beta_{III}$ and $\beta_{I}$ had reached 39.9% and −19.5%, respectively, more than ten times higher than the same values for SB-1. This indicates that the load pattern had a significant effect on the moment redistribution coefficient, β. For this reason, in practical applications it is necessary to consider the effect of the load pattern on the continuous concrete beam in light of the value of β. It is useful to assume a suitable value of β that can ensure the internal force redistribution of the continuous concrete beam, which means enabling the continuous beam to form a sufficient number of plastic hinges to fail from becoming a maneuvering structure.

### 3.4. Strengthening Mechanism under Symmetrical Loading

The two-span continuous beams are statically indeterminate structures, and as such two necessary conditions are needed to determine the internal structural force, namely, the static equilibrium condition and the coordinated deformation conditions. Loading only on one span will result in distinctly different mechanical properties in the structure compared to loading both spans.

**Figure 6:**
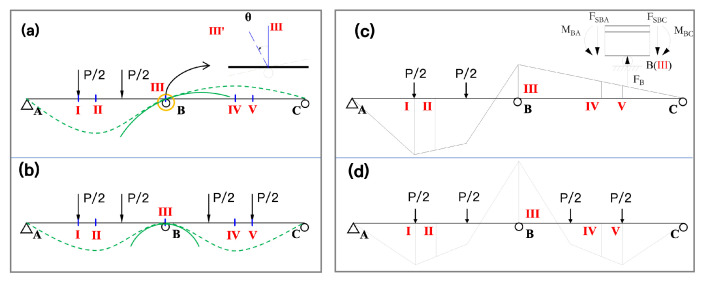
Qualitative deformation of a continuous beam under symmetrical and asymmetrical loading. (**a**) Deformation of the beam SB-1, (**b**) Deformation of the beam SB-2, (**c**) Moment diagram of the beam SB-1, (**d**) Moment diagram of the beam SB-2.

Figure 6 shows the deformation diagram of a continuous beam under symmetrical and asymmetrical loading. This figure shows the mechanical properties of SB-1 and SB-2 and explains the enhancement mechanism of SB-2 under symmetrical loading. Figure 6a illustrates that when span AB is subjected to a vertical downward load, span BC exhibits an upward arching tendency (or arching effect) due to the support at point B, according to the law of the lever. As a result, when span BC is subjected to a load, the load first acts to offset the stress in BC resulting from the arching effect caused by the vertical loading of span AB. In other words, the arching effect can negate some of the vertical load placed on span BC, effectively improving the load-carrying capacity of the span. When spans AB and BC span are simultaneously loaded, each has an arching effect on the other. As a result, the ultimate load ($P_{u}$) of the structure increases and the deformation of the structure decreases.

In recent years, main bridges and the approach spans of bridges have failed due to vehicle overloading, even just a few years after the structures were built [28]. Vehicle loading on these bridges may have been discontinuous due to the use of traffic lights or other interruptions to traffic flow, resulting in an asymmetrically distributed load on the continuous bridges and increasing the likelihood of single-span loading and lower ultimate load of the bridge structure. Therefore, from a safety perspective, bridge designers should carefully examine the mechanical characteristics and the number of steel bars in the middle support section of two-span bridges, examining both the single-span loading and double-span loading simultaneously.

## 4. Finite Element Simulation Calibration

In this paper, the finite element analysis of concrete continuous beams with external prestressed tendons was carried out using the Opensees platform. The OpenSees platform, developed by the University of California, Berkeley, is an open source software framework originally used to develop applications to simulate the performance of structures and geotechnical systems during earthquakes. Today, it is widely used in civil engineering structural modeling for static linear elasticity analysis, static nonlinearity analysis, modal analysis, and dynamic nonlinear analysis. Opensees software has a rich database with high applicability and flexibility, and was able to fully complete the static nonlinear analysis tasks described herein.

**Figure 7:**
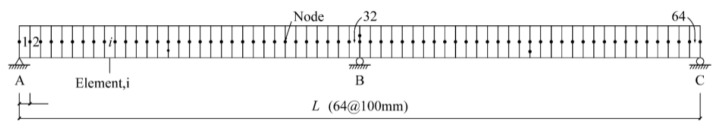
Division of longitudinal elements of continuous beams.

For the finite element model we adopted a two-dimensional three-degrees-of-freedom system, and the analysis plane was an X–Y plane. The model nodes were set along the beam length every 100 mm, and there were 65 nodes in total. Node 1 was a fixed hinge support constraining the x and y directions, while nodes 33 and 65 were rotating hinge supports, constraining the x-direction and applying load by displacement control. The finite element model unit nodes are shown in Figure 7.

The model was divided into two parts: the reinforced concrete beam and the prestressed tendon. The reinforced concrete beams were simulated with the displacement-based beam–column unit (dispBeamColumn), while the prestressed tendons were simulated by truss elements (truss). The prestressed steel bars of unbonded prestressed continuous beams and surrounding concrete could be relatively slipped. The elastic beam element unit (elasticBeamColumn) with sufficient rigidity was used for the connection. The relative sliding between the reinforcement material and the concrete was realized with the equalDOF command. The beam–column unit characteristics were determined by the fiber cross-section. The constitutive model of the concrete adopted Concrete01, and the constitutive model of the prestressed tendon adopted Steel02. The Newton line search algorithm was used to solve the calculation [29].

**Figure 8:**
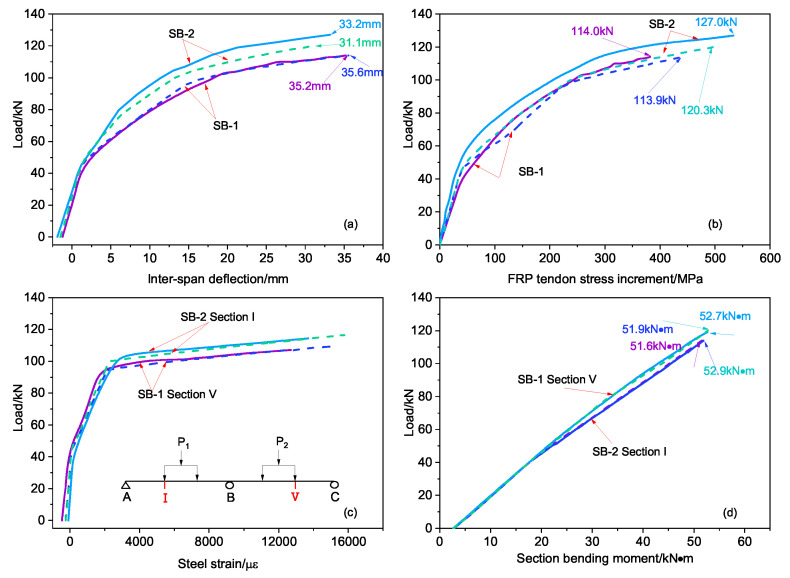
The curve of our group beam between test and FEA results (the dotted line is the simulation value and the solid line is the test value): (**a**) load–span deflection, (**b**) load–external stress increment, (**c**) load–reinforcement strain, (**d**) section bending moment–load.

Figure 8 shows the numerical simulation results for external prestressed CFRP-reinforced concrete continuous beams (SB-1 and SB-2) along with the previous test results. It can be seen that the simulated and measured values of SB-1 and SB-2 in Figure 8c,d are quite consistent; only in the simulation of SB-2 in Figure 8a,b are the values lower than the test data, and these still follow the same trend. The ratio of the simulation value of the ultimate load to the measured value is 0.95, and the ratio of the simulation value of the ultimate deflection to the measured value is 0.94; thus, the difference is within 5%. It can be seen that the load–deflection, load–external tendon stress increment, load–steel tension strain, and load–section bending moment of the test beam are all in good agreement with the measured curve.

## 5. Parametric Study and Results

Many factors affect external prestressed concrete continuous beam performance [30,31]. To find the external prestress tendon increment, the secondary bending moment and bending moment redistribution law, parameters of different tensions, prestress tendon area, eccentricity, and elastic modulus were all studied in combination with the external prestressed steel stranded concrete continuous beam in order to draw useful conclusions.

Twelve rectangular cross-section simulated beams were designed. The parameters of the simulated beams are shown in Table 4. The beam was a two-span external prestressed concrete continuous beam with a span of 12 m, a rectangular cross-section of 600 mm × 300 mm, and a span–depth ratio of 20. The protection layer thickness was 50 mm. The concrete strength was C40. The loading scheme used two-span synchronous single-point loading in the middle of the span.

### 5.1. Stress Increment of External Prestressed Tendons

**Figure 9:**
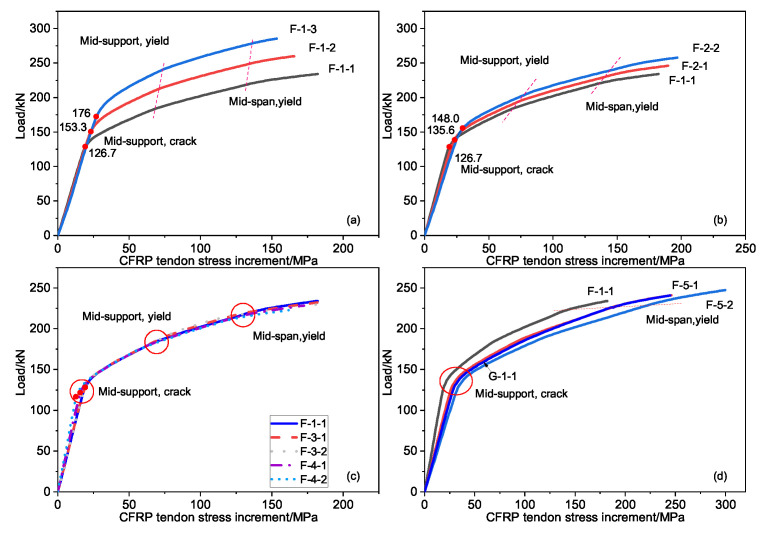
The curve of load and stress increment: (**a**) the initial prestress value the tendons, (**b**) the cross-sectional area of the tendons, (**c**) the cross-sectional area of the reinforcements and the eccentricity of the tendons, (**d**) the elastic modulus of the steel strand and the tendons.

The load–stress increment curves of the external prestressed tendon under different values of prestress, prestressed tendon area, eccentricity, and steel bar cross-sectional area is shown in Figure 9. It can be seen that the stress increment of the external prestressed tendons showed a three-stage development law with an increase in the load value of the continuous beam span, which is consistent with the previous static experiment beam. The occurrence of each critical point originated from the mid-section cracking, the yield of the tensile steel, etc.

The initial prestress value had the greatest influence on the external prestressed CFRP tendon stress increment, followed by the CFRP tendon elastic modulus and the cross-sectional area. The cross-sectional area of the steel bar and the eccentricity of the external prestressed tendons had little effect on the stress increment of the external prestressed tendons when maintaining a constant reinforcement ratio. When the elastic modulus of the steel strand and the external prestressed CFRP tendon were the same, the stress increment of the external prestressed tendon barely changed.

Concerning the influence of the initial value prestress and the tendon cross-sectional area, the critical point (load and external prestress increment) of each stage significantly improved as either the initial prestress or the tendon cross-sectional area increased. In the final failure, the effect of the tendon cross-sectional area on the external prestress increment was consistent with the same critical points, and was inversely affected by the initial prestress, that is, the initial prestress increment decreased when the initial prestress value increased.

### 5.2. Secondary Bending Moments

**Figure 10:**
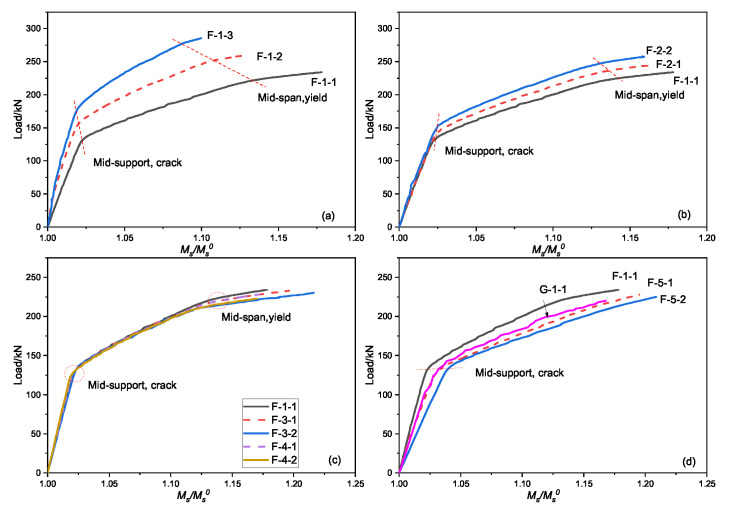
Variation of the secondary bending moment in the mid-support: (**a**) the initial prestress of the tendons, (**b**) the cross-sectional area of the tendons, (**c**) the cross-sectional area of the reinforcements and the eccentricity of the tendons, and (**d**) the elastic module of the steel strand and the tendons.

The relationship of the prestress secondary bending moment and the external load under different factors is shown in Figure 10. Although the signs in Figure 10 are different, there are a few similarities in the trend of the curve. First, the secondary bending moment of the support increases with the load, in keeping with the three-stage development law. Second, the influence of each factor is sequentially reduced by the initial prestress value (Figure 10a), the elastic modulus (Figure 10d), and the cross-sectional area (Figure 10b). Finally, with increasing external prestress CFRP elastic modulus, the load in the middle support section is almost the same at each stage of the prestress secondary bending moment.

**Figure 11:**
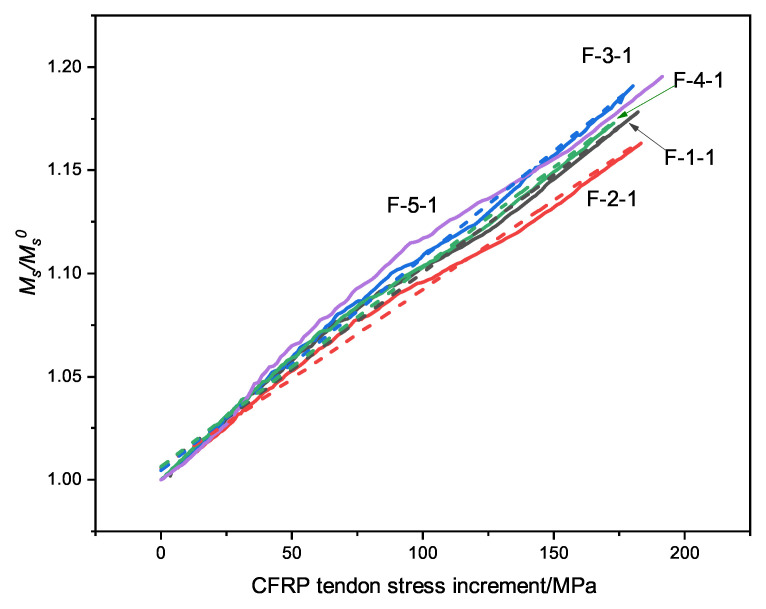
The curve of the secondary bending moment and stress increment.

Figure 11 shows the variation of the stress increment of the external prestressed tendons. The secondary bending moment in the middle support increases with the tendon stress, which has a linear positive correlation. It is not strictly linear, however; in addition to the stress on the CFRP tendons, the change in the continuous beam stiffness has an impact on the secondary bending moment of the continuous beam.

### 5.3. Bending Moment Redistribution

**Figure 12:**
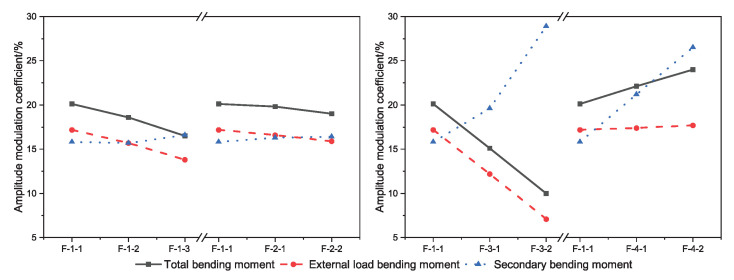
The moment modulation coefficient of the bearing section under ultimate load capacity.

The moment modulation coefficient of the bearing section at the ultimate state is shown in Figure 12. The total bending moment modulation coefficient is between 10.0% and 24.0%, and the external load bending moment modulation coefficient is between 7.1% and 17.7%. The secondary bending moment plays a role in the bending moment modulation; here, the influence degree is between 15.8% and 28.9%. Increasing the axial force of the tendons, reducing the eccentricity, or reducing the number of tensile reinforcements can reduce the modulation coefficient of the total bending moment and the external load bending moment.

The total bending moment amplitude modulation factor of the bearing section of the external CFRP-reinforced concrete continuous beam under the ultimate bearing capacity state can be obtained from Equation (3). However, the total bending moment amplitude modulation includes the influence of the external load bending moment and the secondary bending moment amplitude modulation. In order to more accurately grasp the bending moment redistribution law of the external prestressed CFRP-reinforced concrete continuous beam, the actual bending moment of the external load of the bearing section in the continuous beam is calculated by subtracting the secondary bending moment from the total bending moment.

The external load moment amplitude modulation factor can be calculated as follows:

(4) $$\beta_{load}=\frac{M_{load}}{M_{load}^{e}}$$

The reinforcement coefficient [31] is defined as below:

(5) $$\alpha =\frac{\sigma_{pe}A_{p}+f_{y}A_{s2}-f_{y}^{{}^{\prime }}A_{s2}}{f_{ck}bh_{p}}$$

Table 5 shows the results of the external load moment amplitude modulation coefficient of the simulated beam in its ultimate bearing capacity state. As can be seen, with the exception of Group 3, the external load moment amplitude modulation coefficient of the external prestressed CFRP-reinforced concrete continuous beam decreases with the increase in the comprehensive reinforcement coefficient of the middle bearing section. This is because the area of compression reinforcement was increased in the F-3 group and the area of tensile reinforcement was reduced, leading directly to a decrease in the height of the compression zone [19]. Therefore, the secondary bending moment rose rapidly after the distribution of the reinforcements was changed.

The relationship between the external load bending moment amplitude modulation coefficient and the comprehensive reinforcement index of the middle bearing section can be obtained by data fitting; the equation is as follows:

(6) $$\beta_{load}=-0.554\alpha +0.217$$

Based on the assumption that the secondary bending moment of the middle support section has a linear relationship with the stress increment of the external CFRP reinforcement, the secondary bending moment can be calculated using the following formula:

(7) $$M_{s}=\frac{\sigma_{pu}}{\sigma_{pe}}M_{s0}$$

where $\sigma_{pu}$ is the ultimate stress of the external CFRP bars in the ultimate bearing capacity state, $\sigma_{pe}$ is the effective tensile stress of the external CFRP bars, and $M_{s0}$ is the initial secondary bending moment at the section of the middle support.

By combining Equations (4), (6), and (7), the total bending moment of the bearing section in the externally prestressed CFRP-reinforced concrete continuous beam can be calculated as follows:

(8) $$M=M_{load}+M_{s}=\left(0.554\alpha +0.783\right)M_{load}^{e}+\frac{\sigma_{pu}}{\sigma_{pe}}M_{s0}$$

The SB-2 results and the test results from [31] were selected as the verification data to verify the validity of the proposed formula for calculating the total bending moment of the middle bearing section. Table 6 shows the summary of calculation and test results of the bending moment amplitude modulation formula. The average value of the ratio of the calculated bending moment value of the support section in the obtained continuous beam to the measured bending moment value is 1.04, and the standard deviation is 0.10. The formula proposed in this paper has considerable computational precision. However, the deviation of the calculation results of the SB-2 beam in this paper is relatively large. The reason for this may be that the flexural bearing capacity of the mid-span section of the T-section prestressed concrete continuous beam was correspondingly improved by the existence of the mid-span compression flange.

Through the finite element analysis, the influence of different factors on the load–stress curve was determined and their specific laws of influence identified. This provides a theoretical numerical analysis basis for the design of external prestressed CFRP-reinforced concrete continuous beam structural parameters based on actual engineering conditions and design goals.

## 6. Conclusions

FEA combined with testing was used to study the static behavior of external prestressed CFRP tendon continuous beams. The simulation parameters included the distribution of reinforcements, initial prestress value, section area, and eccentricity of external CFRP tendons. The main conclusions are listed below.

1. The final failure modes of continuous beams SB1 and SB2 are both flexural failure. The crushed concrete in the compression zone causes the continuous beam to lose its bearing capacity.
2. The external prestressed tendon stress increment displays a three-stage development law as external loading increases. The initial prestress value has the greatest influence on the CFRP prestress increment, followed by the elastic modulus and cross-sectional area of the external prestressed tendon. The cross-sectional area of the reinforcements and the eccentricity of the external prestressed tendon has little effect on the stress increment when preserving the same reinforcement ratio. When the elastic modulus of the steel strand and the external prestressed tendon are the same, the external prestressed tendon stress increment hardly changes.
3. The critical point (load and external prestress increment) of each stage significantly improves as the initial prestress value or the tendon cross-sectional area increases. The load at the critical point of each stage is almost the same as the tendon elastic modulus increases. However, the prestress increment of the tendon increases rapidly, showing the opposite influence of the initial prestress and tendon cross-sectional area. The larger the tendon elastic modulus, the larger the external prestress increment during tendon fracture.
4. There is a linear relationship between the secondary bending moment and the tendon stress increment. The distribution of the reinforcements and the eccentricity of the tendons has a large influence on the amplitude and sensitivity of the bending moment. The comprehensive bending moment modulation was between 10% and 24%, and the secondary bending moment modulation accounted for 15.8% and 28.9%.
5. Increasing the axial force of the external prestress tendons or reducing the eccentricity and/or number of tensile reinforcements could reduce the modulation coefficient of the total bending moment and the external load bending moment.
6. We propose a simplified equation for calculating the external load bending moment amplitude coefficient at ultimate. The proposed equation exhibits a quite good fit to test results.

## Figures and Tables

**Table 1:** Properties of steel bars and CFRP tendons.

	Diameter	Yield Strength/MPa	Tensile Strength/MPa	Elastic Modules/GPa
Steel	8	428	604	203
		416	622	196
		441	635	198
	14	419	627	201
		434	613	204
		422	641	197
CFRP	8	-	2052	148
		-	1990	145
		-	2019	142

**Table 2:** The amount of ingredients in the concrete.

Cement	Medium Sand	Gravel	Water
430 kg/m${}^{3}$	460 kg/m${}^{3}$	937 kg/m${}^{3}$	185 kg/m${}^{3}$

**Table 3:** Properties of the concrete.

ID	Compressive Strength/MPa	Elastic Modulus/MPa	Mean Compressive Strength/MPa	Mean Elastic Modulus/MPa
1	27.7	$3.3\times 10^{4}$	27.2	$3.28\times 10^{4}$
2	26.8	$3.25\times 10^{4}$		
3	27.1	$3.27\times 10^{4}$		

**Table 4:** Main variables of simulated beams.

	ID	$\sigma_{\mathrm{pe}}$/MPa	$A_{p}$/${\mathrm{mm}}^{2}$	$A_{s1}$/${\mathrm{mm}}^{2}$	$A_{s2}$/${\mathrm{mm}}^{2}$	*E*/GPa	$h_{p}$/mm
Reference	F-1-1	1000	500	800	400	140	500
	G-1-1	1000	500	800	400	199	500
Initial Prestress	F-1-2	1250	500	800	400	140	500
	F-1-3	1500					
Section area of CFRP tendon	F-2-1	1000	550	800	400	140	500
	F-2-2		600				
Section area of Steal bar	F-3-1	1000	500	700	500	140	500
	F-3-2			600	600		
Eccentricity	F-4-1	1000	500	800	400	140	450
	F-4-2						400
Elastic Module	F-5-1	1000	500	800	400	200	500
	F-5-2					250	

**Table 5:** External load moment amplitude modulation factor.

ID	Reinforcement Coefficient α	$M_{\mathrm{load}}^{e}$	$M_{\mathrm{load}}$	$\beta_{\mathrm{load}}$
F-1-1	0.26	528.32	−437.28	0.172
F-1-2	0.29	586.49	−494.6856	0.157
F-1-3	0.32	644.43	−555.7032	0.138
F-2-1	0.27	555.47	−463.4388	0.166
F-2-2	0.28	581.95	−489.6948	0.159
F-3-1	0.26	525.30	−461.4276	0.122
F-3-2	0.26	535.02	−496.8336	0.071

**Table 6:** Comparison between the calculation results and test results of the bending moment amplitude modulation formula (section of the middle support).

ID	α	$M_{\mathrm{load}}^{e}$	$\sigma_{\mathrm{pe}}$	$\sigma_{\mathrm{pu}}$	$M_{s0}$	$\beta_{\mathrm{load}}$	$M_{\mathrm{calc}}$	$M_{\mathrm{test}}$	$M_{\mathrm{calc}}/M_{\mathrm{test}}$
L1-1A	0.120	−75	560	997	2.94	15.4	−58.48	−59.7	0.98
L2-2B	0.102	−110.5	560	1027	4.41	16.3	−84.68	−86.8	0.98
L3-3C	0.102	−104	550	1003	4.33	16.3	−79.41	−87.6	0.91
L2-3A	0.153	−85	685	1056	5.39	13.9	−65.45	−66.3	0.99
L2-1C	0.183	−96	670	984	3.52	12.5	−79.73	−72.4	1.10
L1-3B	0.277	−78	956	1211	5.02	8.1	−66.68	−58.4	1.14
L3-2A	0.264	−120	876	1117	9.20	8.8	−99.78	−98.9	1.01
SB-2	0.077	−69.58	916	1450	6.9	17.4	−46.53	−37.4	1.24

## Data Availability

Data are available upon request to the corresponding author.
